# Crystal structure of a one-dimensional looped-chain silver(I) coordination polymer: *catena*-poly[[silver(I)-bis­{μ-4-[1-(5′-isopropyl-[1,1′:3′,1′′-terphen­yl]-2′-yl)-1*H*-imidazol-2-yl]pyridine-κ^2^ *N*:*N*′}] nitrate methanol monosolvate monohydrate]

**DOI:** 10.1107/S205698901600949X

**Published:** 2016-06-17

**Authors:** Suk-Hee Moon, Ki-Min Park, Youngjin Kang

**Affiliations:** a Department of Food and Nutrition, Kyungnam College of Information and Technology, Busan 47011, Republic of Korea; b Research institute of Natural Science, Gyeongsang National University, Jinju 52828, Republic of Korea; c Division of Science Education, Kangwon National University, Chuncheon 24341, Republic of Korea

**Keywords:** crystal structure, silver(I), pyridyl­imidazol ligand, looped-chain coordination polymer

## Abstract

In the title compound, {[Ag(C_29_H_25_N_3_)_2_]NO_3_·CH_3_OH·H_2_O}_*n*_, the Ag^I^ cation is four-coordinated by two pyridine N atoms and two imidazole N atoms from four individual 4-(1-(5′-isopropyl-[1,1′:3′,1′′-terphen­yl]-2′-yl)-1*H*-imidazol-2-yl)pyridine (*i*-pro-pyim) ligands. This gives rise to a highly distorted tetra­hedral geometry with bond angles falling in the range 100.33 (19)–122.76 (19)°. Two crystallographically independent *i*-pro-pyim ligands (*A* and *B*) adopt very similar conformations to one another, such that the dihedral angles between the pyridyl and imidazolyl rings in the two ligands are 40.7 (3) and 42.2 (3)°, respectively. Each *i*-pro-pyim ligand binds two symmetry-related Ag^+^ cations, leading to the formation of 14-membered cyclic dimers, in which the Ag^I^ atoms are separated by 6.963 (2) Å for the Ag–*A*_2_–Ag dimer and 7.020 (2) Å for Ag–*B*_2_–Ag. These cyclic dimers are alternately connected to each other by sharing Ag^I^ atoms, resulting in the formation of a looped-chain structure extending along the [100] direction. Moreover, adjacent looped chains are connected by inter­molecular π–π inter­actions [centroid-to-centroid distance = 3.689 (4) Å], giving rise to the formation of a two-dimensional supra­molecular network propagating parallel to (110). Several inter­molecular C—H⋯O and O—H⋯O hydrogen bonds further contribute to the stabilization of the crystal structure.

## Abstract 2

The reaction of Ag^I^ with the pyridyl­imidazol ligand 4-(1-(5′-isopropyl-[1,1′:3′,1′′-terphen­yl]-2′-yl)-1*H*-imidazol-2-yl)pyridine, afforded a nitrate-free one-dimensional looped-chain polymeric structure. The Ag^I^ cation adopts a highly distorted tetra­hedral geometry coordinated by two pyridine N atoms and two imidazole N atoms from four individual ligands.

## Chemical context   

Group-9 metal complexes bearing phenyl­imidazole-based ligands are considered to be suitable triplet emitters for use in phospho­rescent organic light-emitting diodes (PHOLEDs) because of their high efficiency and long-term stability (Cho *et al.*, 2016 ▸). However, there are relatively few reports of the structures of metal complexes that exhibit coordination of pyridyl­imidazole (pyim) ligands with an L-type coordination sphere, which is similar to a phenyl­imidazole system. Recently, Ag^I^ coordination polymers built from pyim ligands have attracted much attention due to their structural diversity and photoluminescence properties which have been shown to depend on the nature of the counter-anion (Lee *et al.*, 2016 ▸). The structural topology of Ag^I^ is quite sensitive to both the counter-anion and solvent mol­ecules (Durá *et al.*, 2014 ▸). Herein, we describe the structure of an Ag^I^ compound with 4-(1-(5′-isopropyl-[1,1′:3′,1′′-terphen­yl]-2′-yl)-1*H*-imidazol-2-yl)pyridine, *i*-pro-pyim, as the pyim ligand. The coordination polymer is obtained by addition of the ligand to AgNO_3_ in methanol/aceto­nitrile. The title nitrate salt is closely related to the perchlorate salt (Lee *et al.*, 2016 ▸).
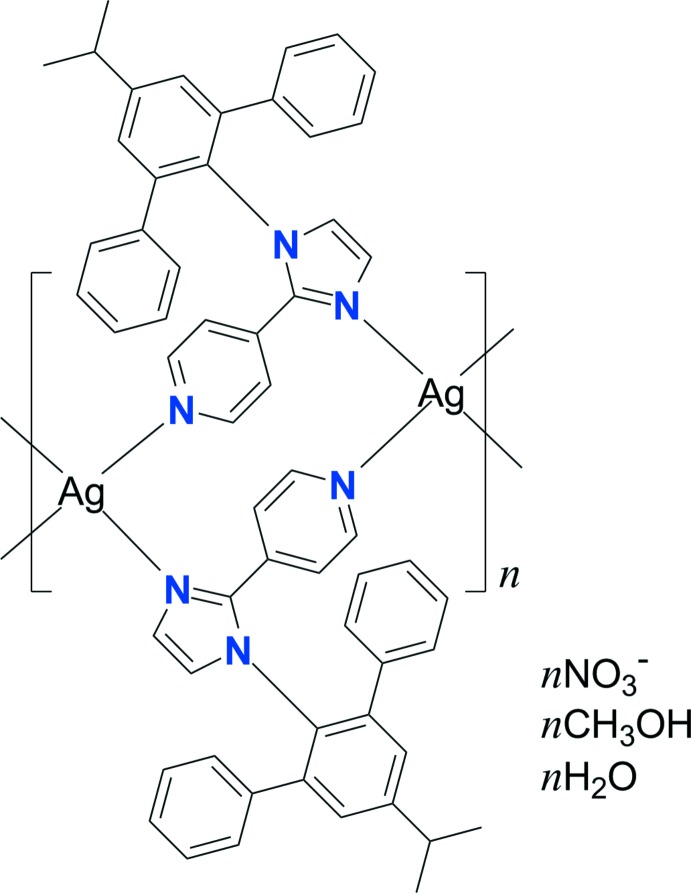


## Structural commentary   

**Figure 1:**
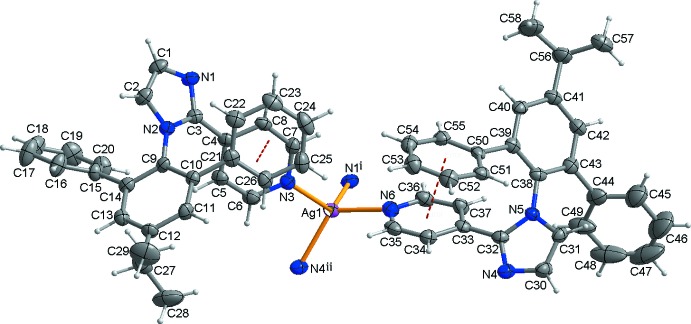
A view of the mol­ecular structure of the title compound with the atom-numbering scheme. The nitrate anion and the lattice solvent mol­ecules have been omitted for clarity. Displacement ellipsoids are drawn at the 30% probability level and red dashed lines represent the intra­molecular π–π inter­actions in the pyim ligand. [Symmetry codes: (i) −*x* + 1, −*y* + 1, −*z* + 1; (ii) −*x* + 2, −*y* + 1, −*z* + 1.]

The title compound crystallizes with one Ag^I^ atom, two pyim ligands (*A* and *B*), one nitrate anion, one methanol solvent mol­ecule, and two water solvent mol­ecules, each with an occupancy factor of 0.5, in the asymmetric unit. As shown in Fig. 1 ▸, the Ag^I^ atom is coordinated by two pyridine N atoms and two imidazole N atoms from four individual *i*-pro-pyim ligands, giving rise to a highly distorted tetra­hedral geometry with bond angles falling in the range of 100.33 (19)–122.76 (19)° (Table 1 ▸). The average Ag—N distance is 2.31 Å, similar to that found in the related perchlorate salt (Lee *et al.*, 2016 ▸).

In the title compound there are two crystallographically independent ligands, *A* and *B*, and their conformations are very similar, such that the dihedral angles between the pyridyl and imidazolyl rings in the two ligands are 40.7 (3) and 42.2 (3)°, respectively. Moreover, there are intra­molecular π–π inter­actions between the pyridyl and phenyl rings of both ligand types, N3,C4–C8 and C21–C26 [centroid-to-centroid distance = 3.760 (4) Å] for *A* and N6,C33–C37 and C51–C56 [centroid-to-centroid distance = 3.716 (4) Å] for *B*.

**Figure 2:**
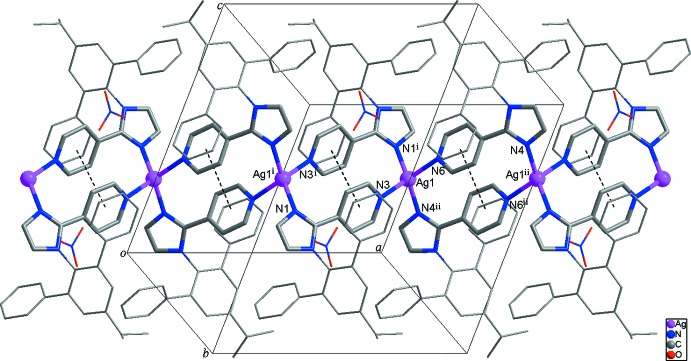
The looped-chain structure of the title compound extending along the *a* axis. The Ag1⋯Ag1^i^ and Ag1⋯Ag1^ii^ distances are 6.963 (2) and 7.020 (2) Å, respectively. Dashed lines represent intra­molecular π–π inter­actions in the looped chain. H atoms and the lattice solvent mol­ecules are omitted for clarity. [Symmetry codes: (i) −*x* + 1, −*y* + 1, −*z* + 1; (ii) −*x* + 2, −*y* + 1, −*z* + 1.]

Two symmetry-related *A* ligands link two Ag^I^ atoms, resulting in the formation of a 14-membered cyclic dimer with an Ag⋯Ag distance of 6.963 (2) Å and a π–π inter­action [centroid-to-centroid distance = 3.890 (4) Å] between N3-containing pyridine rings (Fig. 2 ▸). Similarly, two symmetry-related *B* ligands also connect two Ag^I^ atoms to form another 14-membered cyclic dimer with an Ag⋯Ag separation of 7.020 (2) Å and a π–π inter­action [centroid-to-centroid distance = 3.922 (4) Å] between N6-containing pyridine rings. The two cyclic dimers are connected alternately by sharing Ag^I^ atoms, leading to the formation of a looped-chain structure extending along the *a* axis (Fig. 2 ▸).

## Supra­molecular features   

**Figure 3:**
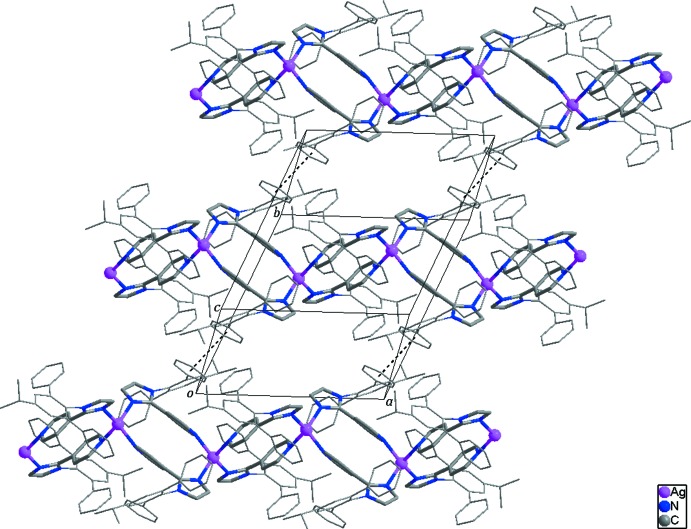
The two-dimensional supra­molecular network formed through inter­molecular π–π inter­actions (dashed lines). H atoms, nitrate anion and the lattice solvent mol­ecules have been omitted for clarity.

Adjacent looped chains in the structure are connected by inter­molecular π–π inter­actions [centroid-to-centroid distance = 3.689 (4) Å] between the C50–C55 and C50^v^–C55^v^ phenyl rings [symmetry code: (v) −*x* + 2, −*y*, −*z* + 1], resulting in the formation of a two-dimensional supra­molecular network propagating parallel to (110) (Fig. 3 ▸). No notable inter­actions are found between the two-dimensional networks. The nitrate anions and lattice solvent mol­ecules occupy the void volume between the layers. The crystal structure of the title compound is further stabilized by weak C—H⋯O hydrogen bonds between the looped chains and the lattice solvent mol­ecules/nitrate anions, and by O—H⋯O hydrogen bonds between the lattice methanol/water mol­ecules or the nitrate anions (Table 2 ▸).

## Synthesis and crystallization   

The *i*-pro-pyim ligand was synthesized according to literature procedures (Lee *et al.*, 2016 ▸). Crystals of the title compound were obtained by combining AgNO_3_ with the *i*-pro-pyim ligand in a 1:1 molar ratio in a mixture of methanol/aceto­nitrile (1:1) and allowing the solution to evaporate slowly at room temperature.

## Refinement   

Crystal data, data collection and structure refinement details are summarized in Table 3 ▸. The anisotropic displacement ellipsoids of some atoms (C53, N7, O1, O2, and O3) were very elongated which indicates static disorder. For these atoms, ISOR restraints were applied (McArdle, 1995 ▸; Sheldrick, 2008 ▸). Two crystallographically independent water O atoms (O1*W* and O2*W*) were refined with site-occupancy factors of 0.5, and their H atoms were not included in the model. All H atoms except those of the water mol­ecules were positioned geometrically and refined using a riding model, with *d*(C—H) = 0.95 Å for C*sp* ^2^—H, 1.00 Å for methine, C—H, 0.98 Å for methyl, and O—H 0.84 Å for hydroxyl H atoms. For all H atoms, *U*_iso_(H) = 1.2–1.5*U*_eq_ of the parent atom.

## Supplementary Material

Crystal structure: contains datablock(s) I, New_Global_Publ_Block. DOI: 10.1107/S205698901600949X/sj5501sup1.cif

Structure factors: contains datablock(s) I. DOI: 10.1107/S205698901600949X/sj5501Isup2.hkl

CCDC reference: 1484735

Additional supporting information: crystallographic information; 3D view; checkCIF report

## Figures and Tables

**Table 1:** Selected geometric parameters (Å, °)

Ag1—N1^i^	2.279 (5)	Ag1—N3	2.306 (5)
Ag1—N4^ii^	2.293 (5)	Ag1—N6	2.330 (6)
			
N1^i^—Ag1—N4^ii^	109.11 (19)	N1^i^—Ag1—N6	100.96 (19)
N1^i^—Ag1—N3	122.76 (19)	N4^ii^—Ag1—N6	121.95 (19)
N4^ii^—Ag1—N3	100.33 (19)	N3—Ag1—N6	103.27 (19)

Symmetry codes: (i) 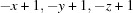; (ii) 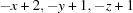.

**Table 2:** Hydrogen-bond geometry (Å, °)

*D*—H⋯*A*	*D*—H	H⋯*A*	*D*⋯*A*	*D*—H⋯*A*
C1—H1⋯O2*W*	0.95	2.49	3.434 (19)	174
C30—H30⋯O2^iii^	0.95	2.47	3.350 (12)	154
C31—H31⋯O4^iii^	0.95	2.36	3.239 (9)	154
O4—H4⋯O3	0.84	2.27	2.846 (16)	126
O4—H4⋯O1*W* ^iv^	0.84	2.30	2.778 (13)	117

Symmetry codes: (iii) 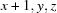; (iv) 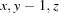.

**Table 3:** Experimental details

Crystal data	
Chemical formula	[Ag(C_29_H_25_N_3_)_2_]NO_3_·CH_4_O·H_2_O
*M*_r_	1050.97
Crystal system, space group	Triclinic, *P* 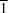
Temperature (K)	173
*a*, *b*, *c* (Å)	13.301 (3), 13.797 (3), 16.527 (3)
α, β, γ (°)	75.919 (13), 71.129 (12), 69.383 (12)
*V* (Å^3^)	2657.3 (10)
*Z*	2
Radiation type	Mo *K*α
μ (mm^−1^)	0.44
Crystal size (mm)	0.13 × 0.12 × 0.10

Data collection	
Diffractometer	Bruker APEXII CCD
Absorption correction	Multi-scan (*SADABS*; Bruker 2013 ▸)
*T*_min_, *T*_max_	0.598, 0.746
No. of measured, independent and observed [*I* > 2σ(*I*)] reflections	38791, 9965, 6302
*R*_int_	0.117
(sin θ/λ)_max_ (Å^−1^)	0.617

Refinement	
*R*[*F* ^2^ > 2σ(*F* ^2^)], *wR*(*F* ^2^), *S*	0.083, 0.208, 1.07
No. of reflections	9965
No. of parameters	658
No. of restraints	30
H-atom treatment	H-atom parameters constrained
Δρ_max_, Δρ_min_ (e Å^−3^)	1.39, −0.88

Computer programs: *APEX2* and *SAINT* (Bruker, 2013 ▸), *SHELXS97* and *SHELXTL* (Sheldrick, 2008 ▸), *SHELXL2014* (Sheldrick, 2015 ▸) and *DIAMOND* (Brandenburg, 2010 ▸).

## supplementary crystallographic information

### Crystal data

**Table:**

[Ag(C_29_H_25_N_3_)_2_]NO_3_·CH_4_O·H_2_O	*Z* = 2
*M**_r_* = 1050.97	*F*(000) = 1092
Triclinic, *P*1	*D*_x_ = 1.314 Mg m^−^^3^
*a* = 13.301 (3) Å	Mo *K*α radiation, λ = 0.71073 Å
*b* = 13.797 (3) Å	Cell parameters from 5559 reflections
*c* = 16.527 (3) Å	θ = 2.4–24.4°
α = 75.919 (13)°	µ = 0.44 mm^−^^1^
β = 71.129 (12)°	*T* = 173 K
γ = 69.383 (12)°	Block, colourless
*V* = 2657.3 (10) Å^3^	0.13 × 0.12 × 0.10 mm

### Data collection

**Table:**

Bruker APEXII CCD diffractometer	6302 reflections with *I* > 2σ(*I*)
φ and ω scans	*R*_int_ = 0.117
Absorption correction: multi-scan (SADABS; Bruker 2013)	θ_max_ = 26.0°, θ_min_ = 1.3°
*T*_min_ = 0.598, *T*_max_ = 0.746	*h* = −16→16
38791 measured reflections	*k* = −17→16
9965 independent reflections	*l* = −20→20

### Refinement

**Table:**

Refinement on *F*^2^	30 restraints
Least-squares matrix: full	Hydrogen site location: inferred from neighbouring sites
*R*[*F*^2^ > 2σ(*F*^2^)] = 0.083	H-atom parameters constrained
*wR*(*F*^2^) = 0.208	*w* = 1/[σ^2^(*F*_o_^2^) + (0.0873*P*)^2^ + 5.0611*P*] where *P* = (*F*_o_^2^ + 2*F*_c_^2^)/3
*S* = 1.07	(Δ/σ)_max_ = 0.001
9965 reflections	Δρ_max_ = 1.39 e Å^−^^3^
658 parameters	Δρ_min_ = −0.88 e Å^−^^3^

### Special details

**Table:**

Geometry. All esds (except the esd in the dihedral angle between two l.s. planes) are estimated using the full covariance matrix. The cell esds are taken into account individually in the estimation of esds in distances, angles and torsion angles; correlations between esds in cell parameters are only used when they are defined by crystal symmetry. An approximate (isotropic) treatment of cell esds is used for estimating esds involving l.s. planes.

### Fractional atomic coordinates and isotropic or equivalent isotropic displacement parameters (Å^2^)

**Table:**

	*x*	*y*	*z*	*U*_iso_*/*U*_eq_	Occ. (<1)
Ag1	0.71139 (4)	0.57116 (4)	0.52858 (3)	0.03240 (18)	
N1	0.3957 (4)	0.4105 (4)	0.3338 (3)	0.0331 (13)	
N2	0.4907 (4)	0.4700 (4)	0.2062 (3)	0.0265 (12)	
N3	0.6602 (4)	0.5156 (4)	0.4312 (3)	0.0321 (13)	
C1	0.3570 (5)	0.3989 (5)	0.2695 (4)	0.0345 (16)	
H1	0.2984	0.3699	0.2790	0.041*	
C2	0.4143 (5)	0.4346 (5)	0.1920 (4)	0.0347 (16)	
H2	0.4041	0.4353	0.1375	0.042*	
C3	0.4762 (5)	0.4539 (5)	0.2935 (4)	0.0253 (14)	
C4	0.5408 (5)	0.4770 (5)	0.3385 (4)	0.0276 (14)	
C5	0.5718 (5)	0.5678 (5)	0.3150 (4)	0.0311 (15)	
H5	0.5535	0.6174	0.2669	0.037*	
C6	0.6300 (6)	0.5842 (5)	0.3635 (4)	0.0353 (16)	
H6	0.6498	0.6473	0.3484	0.042*	
C7	0.6311 (5)	0.4286 (5)	0.4517 (4)	0.0301 (15)	
H7	0.6523	0.3793	0.4990	0.036*	
C8	0.5718 (5)	0.4058 (5)	0.4082 (4)	0.0289 (15)	
H8	0.5523	0.3425	0.4254	0.035*	
C9	0.5755 (5)	0.5037 (5)	0.1370 (4)	0.0251 (14)	
C10	0.6864 (5)	0.4467 (5)	0.1271 (4)	0.0263 (14)	
C11	0.7635 (5)	0.4756 (5)	0.0540 (4)	0.0327 (16)	
H11	0.8400	0.4378	0.0473	0.039*	
C12	0.7329 (5)	0.5578 (5)	−0.0100 (4)	0.0311 (15)	
C13	0.6231 (5)	0.6146 (5)	0.0038 (4)	0.0299 (15)	
H13	0.6016	0.6726	−0.0382	0.036*	
C14	0.5417 (5)	0.5917 (5)	0.0760 (4)	0.0278 (14)	
C15	0.4242 (5)	0.6556 (5)	0.0871 (4)	0.0321 (16)	
C16	0.3730 (6)	0.6716 (6)	0.0228 (5)	0.046 (2)	
H16	0.4142	0.6420	−0.0287	0.055*	
C17	0.2634 (7)	0.7296 (7)	0.0319 (6)	0.060 (2)	
H17	0.2290	0.7394	−0.0129	0.072*	
C18	0.2035 (6)	0.7735 (6)	0.1058 (5)	0.049 (2)	
H18	0.1276	0.8137	0.1123	0.059*	
C19	0.2533 (7)	0.7591 (6)	0.1696 (5)	0.052 (2)	
H19	0.2116	0.7895	0.2207	0.062*	
C20	0.3635 (6)	0.7012 (6)	0.1613 (4)	0.0422 (18)	
H20	0.3977	0.6926	0.2060	0.051*	
C21	0.7238 (5)	0.3561 (5)	0.1921 (4)	0.0300 (15)	
C22	0.6830 (6)	0.2708 (5)	0.2178 (4)	0.0384 (17)	
H22	0.6300	0.2671	0.1925	0.046*	
C23	0.7198 (6)	0.1903 (5)	0.2808 (5)	0.0437 (19)	
H23	0.6919	0.1317	0.2982	0.052*	
C24	0.7957 (7)	0.1952 (6)	0.3178 (5)	0.049 (2)	
H24	0.8190	0.1411	0.3620	0.059*	
C25	0.8386 (6)	0.2790 (6)	0.2910 (5)	0.0428 (18)	
H25	0.8926	0.2816	0.3158	0.051*	
C26	0.8034 (5)	0.3584 (5)	0.2288 (4)	0.0331 (16)	
H26	0.8337	0.4154	0.2105	0.040*	
C27	0.8183 (6)	0.5811 (6)	−0.0920 (5)	0.047 (2)	
H27	0.7774	0.6408	−0.1288	0.057*	
C28	0.9017 (8)	0.6172 (9)	−0.0742 (6)	0.082 (3)	
H28A	0.8628	0.6755	−0.0406	0.124*	
H28B	0.9471	0.5593	−0.0412	0.124*	
H28C	0.9498	0.6404	−0.1290	0.124*	
C29	0.8737 (8)	0.4911 (8)	−0.1437 (6)	0.075 (3)	
H29A	0.8167	0.4696	−0.1541	0.112*	
H29B	0.9215	0.5132	−0.1991	0.112*	
H29C	0.9189	0.4321	−0.1114	0.112*	
N4	1.2737 (5)	0.2683 (4)	0.5446 (4)	0.0344 (13)	
N5	1.2046 (4)	0.1589 (4)	0.6505 (3)	0.0280 (12)	
N6	0.8659 (5)	0.4337 (4)	0.5544 (4)	0.0349 (13)	
C30	1.3587 (6)	0.1915 (5)	0.5721 (4)	0.0362 (17)	
H30	1.4353	0.1868	0.5492	0.043*	
C31	1.3177 (5)	0.1237 (5)	0.6365 (4)	0.0324 (15)	
H31	1.3594	0.0630	0.6664	0.039*	
C32	1.1813 (5)	0.2470 (5)	0.5931 (4)	0.0287 (15)	
C33	1.0702 (5)	0.3098 (5)	0.5830 (4)	0.0281 (15)	
C34	1.0579 (5)	0.3465 (5)	0.4996 (4)	0.0311 (15)	
H34	1.1196	0.3299	0.4510	0.037*	
C35	0.9558 (6)	0.4067 (5)	0.4890 (4)	0.0356 (17)	
H35	0.9480	0.4308	0.4317	0.043*	
C36	0.8792 (6)	0.3971 (5)	0.6346 (4)	0.0367 (17)	
H36	0.8163	0.4146	0.6823	0.044*	
C37	0.9780 (5)	0.3363 (5)	0.6512 (4)	0.0337 (16)	
H37	0.9833	0.3124	0.7090	0.040*	
C38	1.1304 (5)	0.1053 (5)	0.7122 (4)	0.0281 (15)	
C39	1.0600 (5)	0.0720 (5)	0.6869 (4)	0.0280 (15)	
C40	0.9917 (5)	0.0189 (5)	0.7494 (4)	0.0292 (15)	
H40	0.9396	0.0001	0.7330	0.035*	
C41	0.9963 (5)	−0.0081 (5)	0.8356 (4)	0.0326 (16)	
C42	1.0678 (5)	0.0260 (5)	0.8584 (4)	0.0330 (16)	
H42	1.0722	0.0090	0.9166	0.040*	
C43	1.1335 (5)	0.0842 (5)	0.7994 (4)	0.0332 (16)	
C44	1.2032 (6)	0.1244 (7)	0.8286 (4)	0.0449 (19)	
C45	1.2835 (8)	0.0553 (9)	0.8681 (6)	0.071 (3)	
H45	1.2930	−0.0177	0.8769	0.086*	
C46	1.3503 (10)	0.0920 (12)	0.8949 (7)	0.104 (4)	
H46	1.4077	0.0435	0.9194	0.125*	
C47	1.3352 (12)	0.1956 (15)	0.8867 (8)	0.113 (5)	
H47	1.3806	0.2199	0.9063	0.136*	
C48	1.2547 (12)	0.2647 (10)	0.8505 (6)	0.092 (4)	
H48	1.2438	0.3375	0.8452	0.110*	
C49	1.1866 (8)	0.2310 (7)	0.8203 (5)	0.061 (2)	
H49	1.1306	0.2803	0.7948	0.074*	
C50	1.0550 (5)	0.0902 (5)	0.5951 (4)	0.0276 (14)	
C51	1.1497 (6)	0.0635 (5)	0.5277 (4)	0.0314 (15)	
H51	1.2208	0.0346	0.5393	0.038*	
C52	1.1414 (6)	0.0786 (5)	0.4438 (4)	0.0380 (17)	
H52	1.2064	0.0585	0.3982	0.046*	
C53	1.0395 (6)	0.1226 (5)	0.4269 (5)	0.0412 (18)	
H53	1.0341	0.1351	0.3691	0.049*	
C54	0.9447 (6)	0.1488 (5)	0.4930 (5)	0.0381 (17)	
H54	0.8739	0.1784	0.4811	0.046*	
C55	0.9528 (6)	0.1321 (5)	0.5764 (4)	0.0329 (16)	
H55	0.8871	0.1496	0.6220	0.039*	
C56	0.9249 (6)	−0.0709 (6)	0.9032 (5)	0.0447 (19)	
H56	0.8819	−0.0264	0.9500	0.054*	
C57	0.9958 (6)	−0.1685 (6)	0.9451 (5)	0.0470 (19)	
H57A	1.0499	−0.1509	0.9630	0.071*	
H57B	0.9485	−0.1999	0.9958	0.071*	
H57C	1.0354	−0.2185	0.9037	0.071*	
C58	0.8412 (8)	−0.0886 (9)	0.8739 (6)	0.084 (3)	
H58A	0.7983	−0.0219	0.8474	0.126*	
H58B	0.8781	−0.1371	0.8313	0.126*	
H58C	0.7912	−0.1185	0.9234	0.126*	
N7	0.6365 (10)	0.1663 (11)	0.6358 (9)	0.111 (4)	
O1	0.6669 (6)	0.2327 (6)	0.6388 (5)	0.092 (2)	
O2	0.6229 (8)	0.1637 (9)	0.5617 (8)	0.155 (4)	
O3	0.6181 (12)	0.1000 (10)	0.6888 (9)	0.203 (5)	
O4	0.5246 (6)	−0.0588 (6)	0.6947 (4)	0.093 (2)	
H4	0.5785	−0.0525	0.7067	0.139*	
C59	0.5631 (9)	−0.1052 (9)	0.6195 (7)	0.092 (3)	
H59A	0.5676	−0.0510	0.5688	0.138*	
H59B	0.5116	−0.1415	0.6197	0.138*	
H59C	0.6370	−0.1556	0.6175	0.138*	
O1W	0.6345 (13)	0.8346 (9)	0.8223 (9)	0.100 (5)	0.5
O2W	0.1582 (18)	0.2821 (16)	0.2921 (13)	0.147 (7)	0.5

### Atomic displacement parameters (Å^2^)

**Table:**

	*U*^11^	*U*^22^	*U*^33^	*U*^12^	*U*^13^	*U*^23^
Ag1	0.0315 (3)	0.0367 (3)	0.0290 (3)	−0.0147 (2)	−0.0060 (2)	−0.0007 (2)
N1	0.031 (3)	0.039 (3)	0.032 (3)	−0.018 (3)	−0.003 (2)	−0.006 (3)
N2	0.024 (3)	0.029 (3)	0.024 (3)	−0.007 (2)	−0.003 (2)	−0.006 (2)
N3	0.034 (3)	0.035 (3)	0.029 (3)	−0.013 (3)	−0.008 (2)	−0.002 (2)
C1	0.031 (4)	0.041 (4)	0.038 (4)	−0.015 (3)	−0.004 (3)	−0.016 (3)
C2	0.029 (4)	0.043 (4)	0.036 (4)	−0.010 (3)	−0.008 (3)	−0.015 (3)
C3	0.025 (3)	0.024 (3)	0.027 (3)	−0.006 (3)	−0.004 (3)	−0.008 (3)
C4	0.027 (4)	0.031 (4)	0.022 (3)	−0.010 (3)	0.000 (3)	−0.005 (3)
C5	0.036 (4)	0.035 (4)	0.022 (3)	−0.014 (3)	−0.003 (3)	−0.005 (3)
C6	0.042 (4)	0.037 (4)	0.029 (4)	−0.018 (4)	−0.006 (3)	−0.003 (3)
C7	0.027 (4)	0.036 (4)	0.027 (3)	−0.007 (3)	−0.007 (3)	−0.006 (3)
C8	0.026 (4)	0.025 (4)	0.029 (3)	−0.008 (3)	0.000 (3)	−0.002 (3)
C9	0.023 (3)	0.034 (4)	0.020 (3)	−0.009 (3)	−0.004 (3)	−0.008 (3)
C10	0.027 (4)	0.028 (4)	0.024 (3)	−0.007 (3)	−0.006 (3)	−0.006 (3)
C11	0.020 (3)	0.036 (4)	0.036 (4)	−0.002 (3)	−0.005 (3)	−0.006 (3)
C12	0.029 (4)	0.036 (4)	0.025 (3)	−0.012 (3)	−0.002 (3)	−0.001 (3)
C13	0.034 (4)	0.027 (4)	0.030 (3)	−0.006 (3)	−0.011 (3)	−0.005 (3)
C14	0.026 (4)	0.033 (4)	0.026 (3)	−0.009 (3)	−0.004 (3)	−0.010 (3)
C15	0.027 (4)	0.031 (4)	0.035 (4)	−0.002 (3)	−0.010 (3)	−0.007 (3)
C16	0.030 (4)	0.054 (5)	0.051 (5)	0.005 (4)	−0.014 (4)	−0.025 (4)
C17	0.039 (5)	0.074 (6)	0.070 (6)	0.007 (5)	−0.026 (4)	−0.035 (5)
C18	0.030 (4)	0.046 (5)	0.068 (6)	0.003 (4)	−0.012 (4)	−0.023 (4)
C19	0.044 (5)	0.048 (5)	0.044 (5)	0.005 (4)	0.002 (4)	−0.017 (4)
C20	0.044 (5)	0.042 (4)	0.034 (4)	−0.001 (4)	−0.009 (3)	−0.013 (3)
C21	0.024 (4)	0.032 (4)	0.024 (3)	0.000 (3)	0.000 (3)	−0.009 (3)
C22	0.040 (4)	0.033 (4)	0.039 (4)	−0.008 (4)	−0.005 (3)	−0.011 (3)
C23	0.053 (5)	0.026 (4)	0.045 (4)	−0.008 (4)	−0.006 (4)	−0.007 (3)
C24	0.052 (5)	0.038 (5)	0.038 (4)	0.005 (4)	−0.011 (4)	−0.001 (3)
C25	0.037 (4)	0.046 (5)	0.039 (4)	0.001 (4)	−0.016 (3)	−0.006 (3)
C26	0.025 (4)	0.030 (4)	0.037 (4)	−0.001 (3)	−0.007 (3)	−0.006 (3)
C27	0.041 (5)	0.045 (5)	0.040 (4)	−0.009 (4)	0.001 (4)	0.002 (4)
C28	0.080 (7)	0.108 (8)	0.071 (7)	−0.068 (7)	0.008 (5)	−0.013 (6)
C29	0.070 (6)	0.086 (7)	0.048 (5)	−0.026 (6)	0.023 (5)	−0.021 (5)
N4	0.032 (3)	0.031 (3)	0.041 (3)	−0.013 (3)	−0.006 (3)	−0.006 (3)
N5	0.030 (3)	0.023 (3)	0.029 (3)	−0.009 (3)	−0.007 (2)	0.000 (2)
N6	0.038 (3)	0.032 (3)	0.035 (3)	−0.012 (3)	−0.009 (3)	−0.003 (3)
C30	0.025 (4)	0.033 (4)	0.046 (4)	−0.008 (3)	−0.007 (3)	−0.005 (3)
C31	0.022 (4)	0.030 (4)	0.044 (4)	−0.005 (3)	−0.009 (3)	−0.008 (3)
C32	0.031 (4)	0.026 (4)	0.030 (3)	−0.011 (3)	−0.004 (3)	−0.006 (3)
C33	0.034 (4)	0.019 (3)	0.032 (4)	−0.010 (3)	−0.008 (3)	−0.003 (3)
C34	0.027 (4)	0.027 (4)	0.035 (4)	−0.007 (3)	−0.002 (3)	−0.006 (3)
C35	0.051 (5)	0.030 (4)	0.031 (4)	−0.021 (4)	−0.011 (3)	0.000 (3)
C36	0.033 (4)	0.031 (4)	0.035 (4)	−0.007 (3)	0.001 (3)	−0.003 (3)
C37	0.031 (4)	0.030 (4)	0.033 (4)	−0.005 (3)	−0.006 (3)	−0.001 (3)
C38	0.026 (4)	0.021 (3)	0.034 (4)	−0.006 (3)	−0.002 (3)	−0.006 (3)
C39	0.024 (3)	0.023 (3)	0.031 (3)	−0.002 (3)	−0.003 (3)	−0.006 (3)
C40	0.028 (4)	0.023 (3)	0.037 (4)	−0.008 (3)	−0.009 (3)	−0.005 (3)
C41	0.032 (4)	0.028 (4)	0.032 (4)	−0.009 (3)	−0.001 (3)	−0.003 (3)
C42	0.037 (4)	0.028 (4)	0.029 (4)	−0.008 (3)	−0.006 (3)	−0.001 (3)
C43	0.032 (4)	0.029 (4)	0.038 (4)	−0.006 (3)	−0.010 (3)	−0.008 (3)
C44	0.051 (5)	0.062 (5)	0.029 (4)	−0.030 (4)	−0.004 (4)	−0.008 (4)
C45	0.076 (7)	0.094 (8)	0.065 (6)	−0.039 (6)	−0.030 (5)	−0.012 (5)
C46	0.111 (10)	0.151 (13)	0.095 (9)	−0.065 (10)	−0.056 (8)	−0.018 (8)
C47	0.131 (12)	0.192 (16)	0.077 (8)	−0.122 (12)	−0.015 (8)	−0.034 (9)
C48	0.155 (12)	0.113 (10)	0.045 (6)	−0.104 (9)	0.010 (6)	−0.030 (6)
C49	0.094 (7)	0.065 (6)	0.041 (5)	−0.052 (6)	−0.002 (5)	−0.013 (4)
C50	0.036 (4)	0.019 (3)	0.029 (3)	−0.011 (3)	−0.012 (3)	0.002 (3)
C51	0.032 (4)	0.023 (4)	0.036 (4)	−0.010 (3)	−0.007 (3)	0.001 (3)
C52	0.040 (4)	0.034 (4)	0.038 (4)	−0.008 (4)	−0.006 (3)	−0.012 (3)
C53	0.049 (4)	0.039 (3)	0.038 (3)	−0.011 (3)	−0.016 (3)	−0.008 (3)
C54	0.041 (4)	0.029 (4)	0.051 (4)	−0.008 (3)	−0.023 (4)	−0.007 (3)
C55	0.036 (4)	0.030 (4)	0.037 (4)	−0.015 (3)	−0.009 (3)	−0.004 (3)
C56	0.046 (5)	0.054 (5)	0.034 (4)	−0.025 (4)	−0.004 (3)	0.000 (3)
C57	0.055 (5)	0.036 (4)	0.041 (4)	−0.017 (4)	−0.002 (4)	0.001 (3)
C58	0.086 (7)	0.118 (9)	0.068 (6)	−0.075 (7)	−0.023 (6)	0.022 (6)
N7	0.104 (5)	0.109 (5)	0.110 (5)	−0.023 (4)	−0.015 (4)	−0.028 (4)
O1	0.085 (5)	0.083 (5)	0.115 (5)	−0.018 (4)	−0.032 (4)	−0.027 (4)
O2	0.124 (6)	0.170 (8)	0.183 (8)	−0.016 (6)	−0.060 (6)	−0.062 (6)
O3	0.243 (9)	0.132 (7)	0.177 (8)	−0.079 (7)	0.045 (7)	−0.023 (6)
O4	0.080 (5)	0.106 (6)	0.076 (5)	0.004 (4)	−0.031 (4)	−0.015 (4)
C59	0.075 (7)	0.115 (9)	0.073 (7)	−0.010 (7)	−0.006 (6)	−0.036 (7)
O1W	0.152 (14)	0.049 (8)	0.112 (11)	0.005 (8)	−0.106 (11)	0.011 (7)
O2W	0.20 (2)	0.142 (16)	0.138 (16)	−0.106 (16)	−0.062 (15)	0.020 (13)

### Geometric parameters (Å, º)

**Table:**

Ag1—N1^i^	2.279 (5)	N4—Ag1^ii^	2.293 (5)
Ag1—N4^ii^	2.293 (5)	N5—C32	1.355 (8)
Ag1—N3	2.306 (5)	N5—C31	1.367 (8)
Ag1—N6	2.330 (6)	N5—C38	1.434 (8)
N1—C3	1.321 (8)	N6—C35	1.336 (8)
N1—C1	1.384 (8)	N6—C36	1.345 (8)
N1—Ag1^i^	2.279 (5)	C30—C31	1.343 (9)
N2—C3	1.366 (7)	C30—H30	0.9500
N2—C2	1.373 (8)	C31—H31	0.9500
N2—C9	1.440 (7)	C32—C33	1.466 (9)
N3—C7	1.323 (8)	C33—C37	1.379 (9)
N3—C6	1.348 (8)	C33—C34	1.389 (9)
C1—C2	1.338 (9)	C34—C35	1.362 (9)
C1—H1	0.9500	C34—H34	0.9500
C2—H2	0.9500	C35—H35	0.9500
C3—C4	1.454 (8)	C36—C37	1.359 (9)
C4—C5	1.383 (9)	C36—H36	0.9500
C4—C8	1.389 (8)	C37—H37	0.9500
C5—C6	1.381 (9)	C38—C39	1.384 (9)
C5—H5	0.9500	C38—C43	1.411 (9)
C6—H6	0.9500	C39—C40	1.387 (8)
C7—C8	1.375 (9)	C39—C50	1.496 (8)
C7—H7	0.9500	C40—C41	1.398 (9)
C8—H8	0.9500	C40—H40	0.9500
C9—C10	1.382 (8)	C41—C42	1.379 (9)
C9—C14	1.417 (8)	C41—C56	1.525 (9)
C10—C11	1.385 (8)	C42—C43	1.385 (9)
C10—C21	1.490 (9)	C42—H42	0.9500
C11—C12	1.393 (9)	C43—C44	1.477 (10)
C11—H11	0.9500	C44—C45	1.380 (12)
C12—C13	1.365 (9)	C44—C49	1.387 (11)
C12—C27	1.510 (9)	C45—C46	1.388 (13)
C13—C14	1.385 (8)	C45—H45	0.9500
C13—H13	0.9500	C46—C47	1.350 (18)
C14—C15	1.475 (9)	C46—H46	0.9500
C15—C16	1.375 (9)	C47—C48	1.354 (18)
C15—C20	1.388 (9)	C47—H47	0.9500
C16—C17	1.373 (10)	C48—C49	1.409 (13)
C16—H16	0.9500	C48—H48	0.9500
C17—C18	1.372 (10)	C49—H49	0.9500
C17—H17	0.9500	C50—C55	1.380 (9)
C18—C19	1.360 (11)	C50—C51	1.388 (9)
C18—H18	0.9500	C51—C52	1.386 (9)
C19—C20	1.380 (10)	C51—H51	0.9500
C19—H19	0.9500	C52—C53	1.367 (10)
C20—H20	0.9500	C52—H52	0.9500
C21—C22	1.386 (9)	C53—C54	1.377 (10)
C21—C26	1.391 (9)	C53—H53	0.9500
C22—C23	1.393 (10)	C54—C55	1.375 (9)
C22—H22	0.9500	C54—H54	0.9500
C23—C24	1.367 (11)	C55—H55	0.9500
C23—H23	0.9500	C56—C58	1.458 (11)
C24—C25	1.381 (11)	C56—C57	1.503 (10)
C24—H24	0.9500	C56—H56	1.0000
C25—C26	1.371 (9)	C57—H57A	0.9800
C25—H25	0.9500	C57—H57B	0.9800
C26—H26	0.9500	C57—H57C	0.9800
C27—C28	1.496 (11)	C58—H58A	0.9800
C27—C29	1.509 (11)	C58—H58B	0.9800
C27—H27	1.0000	C58—H58C	0.9800
C28—H28A	0.9800	N7—O3	1.137 (14)
C28—H28B	0.9800	N7—O1	1.141 (13)
C28—H28C	0.9800	N7—O2	1.304 (14)
C29—H29A	0.9800	O4—C59	1.400 (11)
C29—H29B	0.9800	O4—H4	0.8400
C29—H29C	0.9800	C59—H59A	0.9800
N4—C32	1.320 (8)	C59—H59B	0.9800
N4—C30	1.364 (8)	C59—H59C	0.9800
			
N1^i^—Ag1—N4^ii^	109.11 (19)	C30—N4—Ag1^ii^	123.4 (4)
N1^i^—Ag1—N3	122.76 (19)	C32—N5—C31	106.8 (5)
N4^ii^—Ag1—N3	100.33 (19)	C32—N5—C38	129.2 (5)
N1^i^—Ag1—N6	100.96 (19)	C31—N5—C38	123.9 (5)
N4^ii^—Ag1—N6	121.95 (19)	C35—N6—C36	116.6 (6)
N3—Ag1—N6	103.27 (19)	C35—N6—Ag1	119.7 (4)
C3—N1—C1	105.7 (5)	C36—N6—Ag1	121.7 (4)
C3—N1—Ag1^i^	124.6 (4)	C31—C30—N4	109.8 (6)
C1—N1—Ag1^i^	123.3 (4)	C31—C30—H30	125.1
C3—N2—C2	106.8 (5)	N4—C30—H30	125.1
C3—N2—C9	130.1 (5)	C30—C31—N5	106.8 (6)
C2—N2—C9	122.6 (5)	C30—C31—H31	126.6
C7—N3—C6	117.3 (6)	N5—C31—H31	126.6
C7—N3—Ag1	120.8 (4)	N4—C32—N5	110.6 (6)
C6—N3—Ag1	119.4 (4)	N4—C32—C33	123.5 (6)
C2—C1—N1	110.0 (6)	N5—C32—C33	125.9 (6)
C2—C1—H1	125.0	C37—C33—C34	118.1 (6)
N1—C1—H1	125.0	C37—C33—C32	123.9 (6)
C1—C2—N2	106.9 (6)	C34—C33—C32	118.0 (6)
C1—C2—H2	126.6	C35—C34—C33	118.8 (6)
N2—C2—H2	126.6	C35—C34—H34	120.6
N1—C3—N2	110.7 (5)	C33—C34—H34	120.6
N1—C3—C4	123.0 (5)	N6—C35—C34	123.8 (6)
N2—C3—C4	126.3 (5)	N6—C35—H35	118.1
C5—C4—C8	118.7 (6)	C34—C35—H35	118.1
C5—C4—C3	122.4 (6)	N6—C36—C37	123.5 (6)
C8—C4—C3	118.9 (6)	N6—C36—H36	118.3
C6—C5—C4	118.0 (6)	C37—C36—H36	118.3
C6—C5—H5	121.0	C36—C37—C33	119.2 (6)
C4—C5—H5	121.0	C36—C37—H37	120.4
N3—C6—C5	123.6 (6)	C33—C37—H37	120.4
N3—C6—H6	118.2	C39—C38—C43	120.6 (6)
C5—C6—H6	118.2	C39—C38—N5	121.1 (6)
N3—C7—C8	123.5 (6)	C43—C38—N5	118.2 (6)
N3—C7—H7	118.3	C38—C39—C40	118.4 (6)
C8—C7—H7	118.3	C38—C39—C50	122.9 (6)
C7—C8—C4	118.9 (6)	C40—C39—C50	118.7 (6)
C7—C8—H8	120.6	C39—C40—C41	122.7 (6)
C4—C8—H8	120.6	C39—C40—H40	118.7
C10—C9—C14	121.1 (6)	C41—C40—H40	118.7
C10—C9—N2	120.5 (5)	C42—C41—C40	117.2 (6)
C14—C9—N2	118.2 (5)	C42—C41—C56	120.3 (6)
C9—C10—C11	118.2 (6)	C40—C41—C56	122.5 (6)
C9—C10—C21	121.7 (5)	C41—C42—C43	122.4 (6)
C11—C10—C21	120.1 (6)	C41—C42—H42	118.8
C10—C11—C12	122.5 (6)	C43—C42—H42	118.8
C10—C11—H11	118.7	C42—C43—C38	118.6 (6)
C12—C11—H11	118.7	C42—C43—C44	120.1 (6)
C13—C12—C11	117.4 (6)	C38—C43—C44	121.3 (6)
C13—C12—C27	121.7 (6)	C45—C44—C49	119.0 (8)
C11—C12—C27	120.8 (6)	C45—C44—C43	119.7 (7)
C12—C13—C14	123.3 (6)	C49—C44—C43	121.2 (8)
C12—C13—H13	118.4	C44—C45—C46	120.3 (11)
C14—C13—H13	118.4	C44—C45—H45	119.9
C13—C14—C9	117.4 (6)	C46—C45—H45	119.9
C13—C14—C15	120.8 (6)	C47—C46—C45	121.1 (13)
C9—C14—C15	121.8 (6)	C47—C46—H46	119.5
C16—C15—C20	118.8 (6)	C45—C46—H46	119.5
C16—C15—C14	119.8 (6)	C46—C47—C48	119.4 (11)
C20—C15—C14	121.4 (6)	C46—C47—H47	120.3
C17—C16—C15	121.1 (7)	C48—C47—H47	120.3
C17—C16—H16	119.5	C47—C48—C49	121.6 (11)
C15—C16—H16	119.5	C47—C48—H48	119.2
C18—C17—C16	119.9 (8)	C49—C48—H48	119.2
C18—C17—H17	120.0	C44—C49—C48	118.6 (10)
C16—C17—H17	120.0	C44—C49—H49	120.7
C19—C18—C17	119.7 (7)	C48—C49—H49	120.7
C19—C18—H18	120.2	C55—C50—C51	118.5 (6)
C17—C18—H18	120.2	C55—C50—C39	119.3 (6)
C18—C19—C20	121.1 (7)	C51—C50—C39	122.1 (6)
C18—C19—H19	119.5	C52—C51—C50	120.5 (6)
C20—C19—H19	119.5	C52—C51—H51	119.8
C19—C20—C15	119.5 (7)	C50—C51—H51	119.8
C19—C20—H20	120.2	C53—C52—C51	119.8 (7)
C15—C20—H20	120.2	C53—C52—H52	120.1
C22—C21—C26	118.8 (6)	C51—C52—H52	120.1
C22—C21—C10	123.3 (6)	C52—C53—C54	120.4 (7)
C26—C21—C10	117.8 (6)	C52—C53—H53	119.8
C21—C22—C23	120.0 (7)	C54—C53—H53	119.8
C21—C22—H22	120.0	C55—C54—C53	119.8 (7)
C23—C22—H22	120.0	C55—C54—H54	120.1
C24—C23—C22	120.3 (7)	C53—C54—H54	120.1
C24—C23—H23	119.9	C54—C55—C50	121.0 (6)
C22—C23—H23	119.9	C54—C55—H55	119.5
C23—C24—C25	119.9 (7)	C50—C55—H55	119.5
C23—C24—H24	120.0	C58—C56—C57	113.9 (7)
C25—C24—H24	120.0	C58—C56—C41	114.8 (6)
C26—C25—C24	120.3 (7)	C57—C56—C41	111.0 (6)
C26—C25—H25	119.9	C58—C56—H56	105.4
C24—C25—H25	119.9	C57—C56—H56	105.4
C25—C26—C21	120.6 (7)	C41—C56—H56	105.4
C25—C26—H26	119.7	C56—C57—H57A	109.5
C21—C26—H26	119.7	C56—C57—H57B	109.5
C28—C27—C29	111.8 (7)	H57A—C57—H57B	109.5
C28—C27—C12	111.4 (7)	C56—C57—H57C	109.5
C29—C27—C12	113.0 (6)	H57A—C57—H57C	109.5
C28—C27—H27	106.7	H57B—C57—H57C	109.5
C29—C27—H27	106.7	C56—C58—H58A	109.5
C12—C27—H27	106.7	C56—C58—H58B	109.5
C27—C28—H28A	109.5	H58A—C58—H58B	109.5
C27—C28—H28B	109.5	C56—C58—H58C	109.5
H28A—C28—H28B	109.5	H58A—C58—H58C	109.5
C27—C28—H28C	109.5	H58B—C58—H58C	109.5
H28A—C28—H28C	109.5	O3—N7—O1	128.4 (17)
H28B—C28—H28C	109.5	O3—N7—O2	115.0 (16)
C27—C29—H29A	109.5	O1—N7—O2	116.6 (14)
C27—C29—H29B	109.5	C59—O4—H4	109.5
H29A—C29—H29B	109.5	O4—C59—H59A	109.5
C27—C29—H29C	109.5	O4—C59—H59B	109.5
H29A—C29—H29C	109.5	H59A—C59—H59B	109.5
H29B—C29—H29C	109.5	O4—C59—H59C	109.5
C32—N4—C30	106.1 (5)	H59A—C59—H59C	109.5
C32—N4—Ag1^ii^	126.3 (4)	H59B—C59—H59C	109.5
			
C3—N1—C1—C2	0.3 (8)	C32—N4—C30—C31	0.5 (8)
Ag1^i^—N1—C1—C2	153.2 (5)	Ag1^ii^—N4—C30—C31	158.6 (5)
N1—C1—C2—N2	−0.2 (8)	N4—C30—C31—N5	−0.6 (8)
C3—N2—C2—C1	0.0 (7)	C32—N5—C31—C30	0.5 (7)
C9—N2—C2—C1	173.1 (6)	C38—N5—C31—C30	177.4 (6)
C1—N1—C3—N2	−0.2 (7)	C30—N4—C32—N5	−0.2 (7)
Ag1^i^—N1—C3—N2	−152.7 (4)	Ag1^ii^—N4—C32—N5	−157.5 (4)
C1—N1—C3—C4	−178.5 (6)	C30—N4—C32—C33	−178.6 (6)
Ag1^i^—N1—C3—C4	29.0 (8)	Ag1^ii^—N4—C32—C33	24.2 (9)
C2—N2—C3—N1	0.1 (7)	C31—N5—C32—N4	−0.2 (7)
C9—N2—C3—N1	−172.2 (6)	C38—N5—C32—N4	−176.9 (6)
C2—N2—C3—C4	178.3 (6)	C31—N5—C32—C33	178.1 (6)
C9—N2—C3—C4	6.0 (10)	C38—N5—C32—C33	1.4 (10)
N1—C3—C4—C5	−140.1 (6)	N4—C32—C33—C37	−138.1 (7)
N2—C3—C4—C5	41.9 (9)	N5—C32—C33—C37	43.8 (10)
N1—C3—C4—C8	39.3 (9)	N4—C32—C33—C34	40.6 (9)
N2—C3—C4—C8	−138.7 (6)	N5—C32—C33—C34	−137.5 (6)
C8—C4—C5—C6	−1.5 (9)	C37—C33—C34—C35	0.0 (9)
C3—C4—C5—C6	177.9 (6)	C32—C33—C34—C35	−178.8 (6)
C7—N3—C6—C5	−0.5 (10)	C36—N6—C35—C34	−0.9 (9)
Ag1—N3—C6—C5	−162.8 (5)	Ag1—N6—C35—C34	163.4 (5)
C4—C5—C6—N3	1.5 (10)	C33—C34—C35—N6	0.6 (10)
C6—N3—C7—C8	−0.4 (9)	C35—N6—C36—C37	0.7 (10)
Ag1—N3—C7—C8	161.5 (5)	Ag1—N6—C36—C37	−163.3 (5)
N3—C7—C8—C4	0.4 (9)	N6—C36—C37—C33	−0.1 (10)
C5—C4—C8—C7	0.6 (9)	C34—C33—C37—C36	−0.2 (9)
C3—C4—C8—C7	−178.8 (5)	C32—C33—C37—C36	178.5 (6)
C3—N2—C9—C10	58.9 (9)	C32—N5—C38—C39	55.6 (9)
C2—N2—C9—C10	−112.3 (7)	C31—N5—C38—C39	−120.6 (7)
C3—N2—C9—C14	−125.8 (7)	C32—N5—C38—C43	−126.5 (7)
C2—N2—C9—C14	63.0 (8)	C31—N5—C38—C43	57.3 (8)
C14—C9—C10—C11	−2.4 (9)	C43—C38—C39—C40	1.0 (9)
N2—C9—C10—C11	172.7 (6)	N5—C38—C39—C40	178.8 (5)
C14—C9—C10—C21	177.9 (6)	C43—C38—C39—C50	−178.7 (6)
N2—C9—C10—C21	−6.9 (9)	N5—C38—C39—C50	−0.8 (9)
C9—C10—C11—C12	−1.3 (10)	C38—C39—C40—C41	−4.3 (9)
C21—C10—C11—C12	178.3 (6)	C50—C39—C40—C41	175.4 (6)
C10—C11—C12—C13	3.7 (10)	C39—C40—C41—C42	4.0 (9)
C10—C11—C12—C27	−174.9 (6)	C39—C40—C41—C56	−177.1 (6)
C11—C12—C13—C14	−2.4 (10)	C40—C41—C42—C43	−0.4 (10)
C27—C12—C13—C14	176.2 (6)	C56—C41—C42—C43	−179.3 (6)
C12—C13—C14—C9	−1.1 (9)	C41—C42—C43—C38	−2.8 (10)
C12—C13—C14—C15	−179.7 (6)	C41—C42—C43—C44	176.1 (6)
C10—C9—C14—C13	3.6 (9)	C39—C38—C43—C42	2.4 (9)
N2—C9—C14—C13	−171.7 (5)	N5—C38—C43—C42	−175.5 (5)
C10—C9—C14—C15	−177.8 (6)	C39—C38—C43—C44	−176.4 (6)
N2—C9—C14—C15	6.9 (9)	N5—C38—C43—C44	5.7 (9)
C13—C14—C15—C16	53.8 (9)	C42—C43—C44—C45	59.3 (10)
C9—C14—C15—C16	−124.8 (7)	C38—C43—C44—C45	−121.9 (8)
C13—C14—C15—C20	−125.6 (7)	C42—C43—C44—C49	−118.0 (8)
C9—C14—C15—C20	55.8 (9)	C38—C43—C44—C49	60.8 (9)
C20—C15—C16—C17	−1.4 (12)	C49—C44—C45—C46	−3.3 (13)
C14—C15—C16—C17	179.2 (7)	C43—C44—C45—C46	179.3 (9)
C15—C16—C17—C18	0.6 (13)	C44—C45—C46—C47	3.1 (17)
C16—C17—C18—C19	0.0 (13)	C45—C46—C47—C48	−1.1 (19)
C17—C18—C19—C20	0.2 (13)	C46—C47—C48—C49	−0.5 (18)
C18—C19—C20—C15	−0.9 (12)	C45—C44—C49—C48	1.7 (11)
C16—C15—C20—C19	1.5 (11)	C43—C44—C49—C48	179.0 (7)
C14—C15—C20—C19	−179.1 (7)	C47—C48—C49—C44	0.2 (14)
C9—C10—C21—C22	55.9 (9)	C38—C39—C50—C55	−130.6 (7)
C11—C10—C21—C22	−123.7 (7)	C40—C39—C50—C55	49.8 (8)
C9—C10—C21—C26	−123.8 (7)	C38—C39—C50—C51	51.3 (9)
C11—C10—C21—C26	56.6 (8)	C40—C39—C50—C51	−128.3 (7)
C26—C21—C22—C23	1.5 (9)	C55—C50—C51—C52	−0.1 (9)
C10—C21—C22—C23	−178.2 (6)	C39—C50—C51—C52	178.0 (6)
C21—C22—C23—C24	0.3 (10)	C50—C51—C52—C53	1.6 (10)
C22—C23—C24—C25	−1.8 (11)	C51—C52—C53—C54	−2.1 (10)
C23—C24—C25—C26	1.4 (11)	C52—C53—C54—C55	1.0 (10)
C24—C25—C26—C21	0.4 (10)	C53—C54—C55—C50	0.7 (10)
C22—C21—C26—C25	−1.8 (9)	C51—C50—C55—C54	−1.1 (9)
C10—C21—C26—C25	177.9 (6)	C39—C50—C55—C54	−179.2 (6)
C13—C12—C27—C28	115.4 (8)	C42—C41—C56—C58	171.2 (8)
C11—C12—C27—C28	−66.0 (9)	C40—C41—C56—C58	−7.7 (11)
C13—C12—C27—C29	−117.7 (8)	C42—C41—C56—C57	−57.8 (9)
C11—C12—C27—C29	60.8 (10)	C40—C41—C56—C57	123.3 (7)

Symmetry codes: (i) −*x*+1, −*y*+1, −*z*+1; (ii) −*x*+2, −*y*+1, −*z*+1.

### Hydrogen-bond geometry (Å, º)

**Table:**

*D*—H···*A*	*D*—H	H···*A*	*D*···*A*	*D*—H···*A*
C1—H1···O2*W*	0.95	2.49	3.434 (19)	174
C30—H30···O2^iii^	0.95	2.47	3.350 (12)	154
C31—H31···O4^iii^	0.95	2.36	3.239 (9)	154
O4—H4···O3	0.84	2.27	2.846 (16)	126
O4—H4···O1*W*^iv^	0.84	2.30	2.778 (13)	117

Symmetry codes: (iii) *x*+1, *y*, *z*; (iv) *x*, *y*−1, *z*.
